# Resident Physicians' Knowledge of Emergency Medical Services: A Comparison Between Emergency Medicine and Non-Emergency Medicine Resident Physicians

**DOI:** 10.7759/cureus.44918

**Published:** 2023-09-08

**Authors:** Michael Cannizzaro, Gavin Kerr, Daniel J Berger, Michael A Dipietro, Jeffrey S Lubin

**Affiliations:** 1 Emergency Medicine, Penn State Health Milton S. Hershey Medical Center, Hershey, USA; 2 Emergency Medicine, Penn State College of Medicine, Hershey, USA

**Keywords:** internal medicine residency, pediatric residency, internal medicine & pediatrics, family medicine residency, emergency medicine training, emergency medicine resident, medical residency, emergency medical services, ems, ems education

## Abstract

Background and objective

Emergency medical services (EMS) are often assumed to only involve bringing patients to physicians for treatment in the emergency department. However, EMS staff are also responsible for responding to physicians in the primary care setting when medical emergencies arise. While emergency medicine (EM) residents are exposed to EMS as part of their curriculum, little is known about the knowledge of other resident physicians who may interact with EMS. In light of this, we conducted this study to address the scarcity of data related to this topic.

Methods

A quantitative cross-sectional knowledge assessment was conducted among resident physicians in emergency medicine, internal medicine, family medicine, pediatric, and combined medicine and pediatric residencies at the Penn State Milton S. Hershey Medical Center.

Results

Eighteen EM residents and 26 non-EM residents completed the assessment. The EM residents had a higher average score when compared to non-emergency medicine residents (69.2% vs. 53.8%, p=0.0012).

Conclusion

Variations in scores between EM and other specialties that interact with EMS highlight the need for further training and familiarization related to EMS for residents in non-EM specialties.

## Introduction

While it is often thought that emergency medical services (EMS) only involve bringing patients to physicians for treatment in the emergency department, EMS staff are also responsible for responding to physicians in the primary care setting when medical emergencies arise. Emergency medicine (EM) residents are exposed to EMS as part of their curriculum; however, little is known about the knowledge of other resident physicians who may interact with EMS.

EMS providers have unique skills and expertise that significantly contribute to patient care when used appropriately in out-of-hospital settings. EMTs and paramedics have the ability to evaluate and treat a wide range of health conditions in diverse settings and have specific training in cardiology, pulmonology, and trauma [1]. Research on the association between physicians’ knowledge of EMS and the quality of patient care is limited. However, studies have shown that higher-performing hospitals have stronger collaborative relationships with EMS personnel [2]. For example, it has been found that hospitals that prioritize bidirectional communication with EMS and adopt shared patient-centered missions achieve lower myocardial infarction mortality rates. This study aims to investigate and compare the current level of EMS-related knowledge between emergency medicine resident physicians and residents of other specialties that regularly interact with EMS. As per the Accreditation Council for Graduate Medical Education (ACGME), EMS is a core competency of emergency medicine, and documented experience with EMS is required for the completion of an emergency medicine residency [3]. However, this is not a requirement for the other specialties included in this study; hence, it was hypothesized that emergency medicine residents would have a greater understanding and knowledge related to EMS than residents of other specialties due to this EMS education requirement.

## Materials and methods

An online knowledge assessment model was employed to assess resident physicians' knowledge of the EMS system. Residents at the Penn State Milton S. Hershey Medical Center in emergency medicine, internal medicine, family medicine, pediatric, and combined medicine and pediatric residencies were recruited into the study via emails from their chief residents and/or program coordinators. Study data were collected and managed using REDCap (Research Electronic Data Capture) tools hosted at Pennsylvania State University [4,5]. REDCap is a secure, web-based software platform designed to support data capture for research studies, providing (1) an intuitive interface for validated data capture; (2) audit trails for tracking data manipulation and export procedures; (3) automated export procedures for seamless data downloads to common statistical packages; and (4) procedures for data integration and interoperability with external sources.

The survey consisted of 14 multiple-choice questions, which were formulated by members of the research team, which included a board-certified EMS physician. One question was excluded from the analysis after responses were collected due to multiple potentially correct answers. Data were analyzed in Microsoft Excel (Microsoft, Redmond, WA). Descriptive statistics were utilized to analyze respondent demographic data and the Fisher’s exact test was used to compare EM and non-EM resident performance on specific questions. T-tests were employed to compare average assessment scores between EM and non-EM residents.

## Results

A total of 44 resident physicians completed the assessment. The number of respondents and average score by specialty are shown in Table 1. Emergency medicine residents had a mean score of 69.2% with regard to correct responses, higher than the average score of non-emergency medicine residents (53.8%) (p=0.0012).

**Table 1:** Number of completed questionnaires and average scores for each residency

	Emergency medicine	Family medicine	Internal medicine	Pediatric	Combined internal medicine/pediatric
Number of completed questionnaires	18	5	8	9	4
Average score	70%	58%	47%	54%	62%

As seen in Table 2, the highest-scoring question was related to whom EMS providers contacted while transporting or on the scene. The lowest-scoring questions were related to what medication EMS providers could administer during intubation and who determines what level of EMS provider was dispatched to the scene of a medical emergency. It is important to note that there was a wide range of scores for individual questions across all specialties. For instance, 78% of emergency medicine residents were aware that EMS providers can provide medical therapies only when ordered by command-certified physicians within their chain of command, while 60% of family medicine physicians believed that physicians on the scene could also order EMS providers to provide medical therapies. Additionally, 89% of emergency medicine residents understood that an Advanced Life Support license was necessary to start an IV in our state, while 75% of internal medicine residents assumed that Basic Life Support providers could start an IV.

**Table 2:** Percentage of correct responses for each residency by question EMS: emergency medical services; EMT: emergency medical technician; MVC: motor vehicle crash

Question	Emergency medicine (n=18)	Family medicine (n=5)	Internal medicine (n=8)	Pediatric (n=9)	Combined internal medicine/pediatric (n=8)
1. What EMS provider can start an IV?	89%	100%	25%	67%	75%
2. EMS providers must provide medical therapies when ordered by	78%	40%	38%	67%	50%
3. For intubation in the field, the EMS crew can administer	44%	40%	13%	22%	25%
4. After a patient has been intubated in the field for prolonged transport, EMS can	78%	60%	75%	89%	100%
5. Who determines what level of EMS provider will be dispatched to the scene?	33%	40%	25%	11%	50%
6. Do physician providers ever respond to the scene?	94%	60%	38%	33%	25%
7. Throughout the United States, EMS systems are owned by	94%	100%	88%	89%	100%
8. What is the average number of training hours required by paramedics to qualify for certification?	44%	20%	0%	33%	25%
9. Once arriving at the scene with multiple people injured from an MVC, what is the first responsibility of the EMS provider?	61%	60%	63%	78%	100%
10. Whom do EMS providers contact if they have medical questions while transporting or on scene?	100%	100%	100%	89%	100%
11. If you as a physician are involved in patient care at the scene and provide medical orders to the EMS team, you should	56%	20%	38%	33%	50%
12. Are all EMTs always operating under the direction of a physician?	44%	40%	13%	22%	25%
13. What level of EMS services is required to administer oxygen therapies?	89%	80%	100%	67%	75%

## Discussion

This study's findings reveal that emergency medicine residents have more knowledge of EMS than non-emergency medicine residents. This is not surprising since EMS education is part of emergency medicine's core competency per ACGME, while the same is not true for non-emergency medicine residencies. A lack of knowledge about the capabilities and limitations of EMS providers among physicians could potentially impact the quality of patient care. This may result in ineffective collaboration between physicians and EMS providers, leading to difficulties in developing shared treatment plans for patients. Furthermore, miscommunications between physicians and EMS providers could negatively impact patient safety; effective communication between EMS and emergency departments could potentially prevent patient harm [6].

The EMS Educators Committee of the Society of Teachers of Emergency Medicine and the University Association for Emergency Medicine have proposed a model curriculum aimed at standardizing EMS education for emergency medicine residents. The goal of the curriculum was to develop familiarity with the design and operation of prehospital EMS systems and provide experience in prehospital emergency care [7]. The authors speculate that the prevailing inconsistency may be due to insufficient distribution or shortcomings of the previously proposed EMS curriculum. To ensure that all specialties have a basic understanding of EMS, expanding this curriculum to not only emergency medicine but also other specialties may be beneficial.

Although this study is the first to compare the knowledge related to EMS among residents of different specialties, it has several limitations that could impact the generalizability of the findings, including its small sample size and the fact that it was a single-center study. Therefore, the results could be only reflective of our institution’s EMS education and not generalizable to other regions or hospitals. Additionally, participation was voluntary, and hence the sample could have been affected by self-selection bias. Also, the cross-sectional design limited the ability to observe changes in knowledge over the course of a residency; hence, it is possible that residents may accrue more knowledge about EMS over time. The form also did not track years of residency or prior EMS experience, both additional sources of bias that could potentially skew the data. Lastly, the questions used in the assessment were designed by the research team and were not from a validated instrument.

Future research would benefit from a larger sample size that includes participants from various medical schools and regions. Researchers should consider using a stratified random sampling technique with incentives for participation to help mitigate bias. Additionally, the study could be replicated with attending physicians who have completed training, including any EMS curriculum if available, rather than residents.

## Conclusions

Knowledge of EMS is higher among emergency medicine residents compared to their non-emergency medicine counterparts. Although physicians outside of the emergency medicine specialty interact with EMS less commonly, a lack of basic understanding of the capabilities of EMS providers may negatively impact patient handoff due to miscommunication, incorrect assumptions about the capabilities of the EMS providers, or misunderstandings about the medical command structure. This could be addressed by providing basic education on the role and scope of EMS practice to non-emergency medicine residents.
